# Expression of the oncogenic, constitutive active Platelet-Derived Growth Factor Receptor alpha D842V mutant induces loss of primary cilia

**DOI:** 10.1186/2046-2530-4-S1-P63

**Published:** 2015-07-13

**Authors:** BS Nielsen, F Schmid, R Malinda, ST Christensen, LB Pedersen

**Affiliations:** 1 Department of Biology, University of Copenhagen, Copenhagen, Denmark

## Objective

The receptor tyrosine kinase (RTK) Platelet-Derived Growth Factor Receptor α (PDGFRα) localizes to the primary cilium, and expression of the constitutive active PDGFRα D842V mutant has been linked to gastrointestinal tumor formation. We set out to investigate whether expression of D842V causes loss of primary cilia in cultured cells.

## Methods

Plasmids encoding GFP-tagged wild type and D842V mutant PDGFRα were expressed in hTERT-RPE1 cells. To promote ciliogenesis, transfected cells were grown under serum-free conditions for 12 hours and in the presence of various inhibitors. Cilia numbers of GFP-positive cells were assessed by immunofluorescence microscopy with antibodies against cilia markers. In parallel, cells were analyzed by western blotting to evaluate expression level and activity of the tagged receptors.

## Results

Cells expressing the D842V mutant contained significantly fewer cilia than cells expressing wild type PDGFRα. Cilia numbers of D842V-expressing cells were restored to control levels upon treatment with the RTK inhibitor crenolanib, but not with other RTK inhibitors such as AG1296 or imatinib. Western blot analysis confirmed that D842V activity is inhibited by crenolanib, but not by AG1296 and imatinib. Treatment with inhibitors against AKT, ERK1/2 and HDAC6 failed to restore cilia numbers in D842V-expressing cells, suggesting that the D842V mutant induces cilium loss by an alternate pathway.

## Conclusions

Our results indicate that the D842V mutant promotes loss of primary cilia by a mechanism that depends on its kinase activity, but not on activation of AKT, ERK1/2 or HDAC6. We are currently investigating the mechanism by which D842V induces loss of cilia.

